# Shelf‐life extension of whole shrimp using an active coating containing fish skin gelatin hydrolysates produced by a natural protease

**DOI:** 10.1002/fsn3.1293

**Published:** 2019-12-03

**Authors:** Armin Mirzapour‐Kouhdasht, Marzieh Moosavi‐Nasab

**Affiliations:** ^1^ Seafood Processing Research Group School of Agriculture Shiraz University Shiraz Iran; ^2^ Department of Food Science and Technology School of Agriculture Shiraz University Shiraz Iran

**Keywords:** actinidin, active coating, gelatin hydrolysates, shelf‐life

## Abstract

This study was focused on shelf‐life extension of whole shrimp (*Penaeus merguiensis*) using an active coating containing gelatin hydrolysates. Gelatin extracted from *Scomberomorus commerson* skin was hydrolyzed using actinidin extracted from kiwifruit. Some important physicochemical characteristics of fish skin gelatin including viscosity, gelling and melting points, and temperatures were examined. The whole shrimp was coated with four coating agents including fish skin gelatin (FG), commercial gelatin (CG), fish skin gelatin containing 1 mg/ml fish gelatin hydrolysates (FG + GH), and commercial bovine gelatin containing 1 mg/ml fish gelatin hydrolysates (CG + GH). Chemical, microbial, and sensorial properties of samples were monitored for 12 days at 4°C with 3‐day intervals (0–12 days). The pH value of samples coated with FG + GH and CG + GH showed the lowest changes during 12 days of storage (1.68 ± 0.00 and 1.70 ± 0.09, respectively). The free fatty acid content (FFA), total volatile base nitrogen (TVB‐N), lipid oxidation, and carbonyl content of samples coated with FG + GH and CG + GH were significantly lower than that of control, CG, and FG samples. The results of this study showed that the gelatin hydrolysates could be used as a preservative costing agent for whole shrimp.

## Graphical abstract

The gelatin hydrolysates were used as an active agent for whole shrimp active coating. The physicochemical, microbial, and sensorial characteristics of shrimp were improved after coating with these hydrolysates.

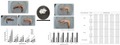

## 1 INTRODUCTION

Gelatin, a colloidal protein with unique structure and functional properties, is obtained by partial hydrolysis of collagen which is the most abundant protein of the skin, bones, and connective tissues of animals (Mirzapour‐Kouhdasht, Sabzipour, Taghizadeh, & Moosavi‐Nasab, 2019). Gelatin has shown increasingly food and pharmaceutical functionality as a result of its specific nature (Gómez‐Guillén et al., 2009; Kouhdasht, Moosavi‐Nasab, & Aminlari, 2018). The high‐molecular‐weight polypeptide chains in gelatin lead to potent film‐forming capacity (Zhang, Dutilleul, Li, & Simpson, 2019). The FG‐based coatings and films enhance food quality by extending their shelf life. This gelatin with capability of producing a transparent film could be a suit delegation (Hosseini & Gómez‐Guillén, 2018). Fish gelatin could be used as a well‐defined carrier for active agents like curcumin/β‐cyclodextrin in order to extend the shelf life by reducing chemical and microbial corruption (Sun et al., 2019). High potential of marine products (e.g., shrimp in this study) to changes caused by biochemical reactions and microbial metabolism result in deterioration of their quality (Yu, Regenstein, & Xia, 2019). Microbial growth is the major cause for the spoilage of most seafood products (Han, Ruiz‐Garcia, Qian, & Yang, 2018). Bioactive compounds are promising components for food packaging (Hosseini & Gómez‐Guillén, 2018). Gelatin‐specific structure slows down the spoilage of food products by controlling the release of the bioactive compounds from the film to the coated food during the storage (Abdelhedi et al., 2019).

The *Scomberomorus commerson* has not been commercialized. Furthermore, the utilization of wastes of this fish for gelatin and bioactive peptides production could be a promising way for valorization.

Different studies have proved that the gelatin hydrolysates from salmon, cod, hoki, pollack, snapper, and sole exhibit antioxidant and iron‐chelating activities (Byun & Kim, 2001; Giménez, Alemán, Montero, & Gómez‐Guillén, 2009; Guo et al., 2013; Phanturat, Benjakul, Visessanguan, & Roytrakul, 2010). Antimicrobial effect of gelatin peptides, another characteristic which is very important for preparing coating agents, is also reported by various researches (Abdelhedi et al., 2017; Guillén et al., 2010; Kouhdasht & Moosavi‐Nasab, 2018; Lima et al., 2015; Yi, De Gobba, Skibsted, & Otte, 2017). Among different methodologies for extending the shelf life of refrigerated seafood products, coating by biopolymers is mainly the method of choice (Moosavi‐Nasab, Mirzapour‐Kouhdasht, & Oliyaei, 2019).

Recently, a few studies have investigated the effect of gelatin incorporated with bioactive peptides on shelf life of marine products. The goal of this research was to survey the effect of four coating strategies including CG, FG, CG + GH, and FG + GH on the shelf life of whole shrimp (*Penaeus merguiensis*) during 12 days of storage at refrigerator temperature. For this purpose, the gelatin extracted from *Scomberomorus commerson* skin was hydrolyzed using plant protease (actinidin) and combined with fish and commercial bovine gelatin‐coating solutions in order to investigate the chemical properties such as pH, FFA, TVB‐N, thiobarbituric acid reactive substances (TBARS), and protein oxidation were examined alongside with microbial, and sensory analyses.

## 2 MATERIALS AND METHODS

### 2.1 Materials

Banana prawn shrimp (*Penaeus merguiensis*) was provided by Bandar Abbas fishing pier, Iran. Barred mackerel (*Scomberomorus commerson*) skins were provided from Shiraz fish market, Shiraz, Iran. All the chemicals and reagents were analytical grade and provided from Merck Co. Actinidin protease (175.28 U/mg) was obtained from our last works at Seafood Processing Research Group, Shiraz University, Shiraz, Iran.

### 2.2 Gelatin extraction and hydrolysis

Gelatin was extracted using hot water method after swelling with slight modifications (Duan, Zhang, Liu, Cui, & Regenstein, 2018). Briefly, after the skins were soaked in 0.1 N NaOH solution at a ratio of 1:5 (w/v) for 6 hr, they were washed with distilled water until neutral pH. The skins were soaked in distilled water at a ratio of 1:10 (w/v) and heated at 65°C for 6 hr. Gelatin powder was obtained after centrifuging (5,000 × *g*, 30 min) followed by freeze‐drying the supernatant with a yield of 21.04%.

Gelatin powder was hydrolyzed using actinidin protease. Summarily, gelatin solution (2.5% w/v) in Tris–HCl buffer (pH was adjusted to 7.5) hydrolyzed by addition of 50 U/mg actinidin (stirred at 80 rpm, 37°C, 6 hr). The pH was maintained at 7.5 during the hydrolysis using 0.1 N NaOH. Subsequently, the suspension was heated at 85°C for 15 min followed by centrifuging at 8,000 × *g* for 15 min at 10°C. The supernatant was freeze‐dried and kept at −20°C until further experiments. All the experiments were performed in triplicate.

### 2.3 Degree of hydrolysis determination

The degree of hydrolysis (DH) was determined by TNBS method (Adler‐Nissen, 1979). Aliquots of samples were dissolved in 2 ml of sodium phosphate buffer (0.2125 M, pH 8.2). 0.1% TNBS solution (2 ml) was poured to the mixture before incubation at 50°C for 60 min. Afterward, 4 ml HCl (0.1 N) was added to stop the reaction. The samples were kept at ambient temperature for 30 min followed by measuring of absorbance at 340 nm. A solution of 0–2 mM L‐Leucine was used for standard curve. The DH was calculated by dividing the difference between the amino nitrogen content of the protein substrate after and before hydrolysis (mg/g protein) into the nitrogen content of the peptide bonds in the protein substrate (Npb). The Npb of fish gelatin is 155.5 (Adler‐Nissen, 1979).

### 2.4 Gelatin characteristics

#### 2.4.1 Viscosity

The viscosity (cP) of samples was measured using Brookfield digital viscometer (Brookfield DV‐II + Pro) with No. 1 spindle (Ki et al., 2005). Analyses were performed in triplicate at 25 ± 0.5°C.

#### 2.4.2 Gelling and Melting temperature and time

The gelling temperature and time were determined as described by Muyonga, Cole, and Duodu (2004) with some modifications (Muyonga et al., 2004). Analyses were performed in triplicate.

### 2.5 Preparation of coatings and coating operation

Shrimps with average weight of 20 ± 1 g were rinsed with tap water and kept at 4°C until coating operation. Gelatin solutions (CG and FG) with a concentration of 4% (w/v) was prepared at 45°C with constant stirring. Another coating containing GH was provided by adding 1 mg/ml of GH to the FG and CG solutions. The whole shrimps were dipped in every solution (CG, FG, FG + GH, and FG + GH) for 30 s twice with 2‐min interval. Afterward, samples were kept at 4°C for 30 min in order to complete coating process. Samples were immediately packed in polyethylene bags and returned to 4°C until further experiments at 0, 3, 6, 9, and 12 days.

### 2.6 Chemical characterization of the coated shrimp

#### 2.6.1 Evaluation of pH

The pH of solutions (10% w/v) was measured using a pH meter (Laboratory pH meter, PHT 110).

#### 2.6.2 Free fatty acid determination

The oil extracted from the samples using cold pressing (5 g) was mixed with 25 ml of ethanol, following by addition of few drops of 1% phenolphthalein. The samples were then titrated by 0.1 N NaOH until pink color achieved (the color must be stable at least 30 s). The free fatty acid (FFA) content was determined in terms of oleic acid using the following equation:

$$\text{FFA}\;(\%)\;=\;\;\frac{282\;\times \;N\;\times \;100\;\times \;V}{1000\;\times \;W}$$

where *N* is the normality of NaOH, *V* is the volume of NaOH, *W* is the sample weight, and 282 is molecular weight of oleic acid (Horwitz, 2000).

#### 2.6.3 Total volatile basic nitrogen

This experiment was performed using micro Kjeldahl distillation method. Minced samples (10 g) were distilled and collected in a flask containing 2% boric acid and few drops of methyl red. Subsequently, the solution was titrated using 0.1 N sulfuric acid until color changed to purple. The volume of consumed sulfuric acid was multiplied by 14 to calculate the total volatile basic nitrogen (mg N/100 g shrimp sample) (Abelti, 2013).

#### 2.6.4 Lipid oxidation secondary products

The oxidation secondary products of lipids were determined by evaluation of TBARS (Lee, Jiang, Brenna, & Abbaspourrad, 2018). The amounts of TBARS of samples were calculated by the following equation:

$$\text{TBARS}\;=\;50\;\times \;[\left(A_{\mathrm{sample}}\;-\;A_{\mathrm{control}}\right)/200]$$

#### 2.6.5 Determination of protein oxidation

The protein oxidation was calculated by measuring the carbonyl content (Vuorela et al., 2005). The standard curve was achieved using bovine serum albumin (BSA). The carbonyl content was calculated according to the following equation:

$$\text{Carbonyl}\;\text{content}\;=\;A_{370}\;/\;21$$

where 21 is the specific molar extinction coefficient of carbonyls in terms of mM^−1^ cm^−1^.

### 2.7 Microbial analysis

Serial dilutions were provided, and total microbial count was measured by pour plate method on the PCA culture medium. In order to count the mesophilic aerobic bacteria (MAB), the plates were incubated at 37°C for 48 hr after inoculation. The psychrotrophic bacteria (PB) were determined by incubating the plates at 7°C for 10 days. The lactic acid bacteria (LAB) were also cultured on MRS agar medium and incubated in anaerobic conditions at 25°C for 5 days (Raeisi, Tajik, Aliakbarlu, Mirhosseini, & Hosseini, 2015).

### 2.8 Sensorial analysis of coated shrimp

The sensory analysis was performed by a 5‐member panelist group (Ruiz‐Cruz et al., 2010). Three pieces of every batch were given to the panelists with knowledge of sensorial evaluation methods in a similar condition of light, air temperature, and humidity. After taking apart in complementary sections, a 5‐point hedonic method was used to score the color, odor, texture, and total acceptance from 1 (dislike very much) to 5 (like very much). Fish fillets were considered to be acceptable when the obtained score was ≥3.

### 2.9 Statistics

Statistical analysis of the results was operated using the statistical software SPSS ver. 21.0. The one‐way and two‐way analysis of variance (ANOVA) was performed with the Tukey test to determine the significance among the mean comparisons at the probability level of 95%.

## 3 RESULTS AND DISCUSSION

### 3.1 Gelatin characteristics

#### 3.1.1 Viscosity

This characteristic is directly affected by molecular weight, polydispersity, and gel strength of the gelatin (Mirzapour‐Kouhdasht et al., 2019). The viscosity of extracted gelatin in the present study (7.52 cP) was not significantly higher than that of commercial bovine gelatin (7.46) (*p* < .05). The higher value of viscosity in FG was probably due to the breakdown of electrostatic and hydrogen bonds during the pretreatment and main treatment of gelatin production. The viscosity of FG + GH and CG + GH solutions was 6.21 and 6.29 cP, respectively, which significantly lower than gelatin solutions.

#### 3.1.2 Gelling and melting points

Hydroxyproline and proline are responsible for the stability of the structure through the hydrogen bond with the free water molecules (Mirzapour‐Kouhdasht et al., 2019; Muyonga et al., 2004). The gelling temperature of Rohu and common carp skin gelatins has been reported as 18.52 and 17.96°C, respectively (Ninan, Jose, & Abubacker, 2011). In the present study, the formation temperature of gel for FG was 19.95°C, which showed little difference with the results of CG (19.44°C) and previous studies. The time required to form gelatin gels of Rohu and common carp skin gelatins was reported to be 106 and 103 s, respectively, while the gelling time of *Scomberomorus commerson* skin gelatin extracted in the same research was 104.51 s (Ninan et al., 2011).

The melting temperature of the *Scomberomorus commerson* skin gelatin and commercial bovine gelatin was 23.10°C and 25.36°C, respectively. The melting time of *Scomberomorus commerson* skin and commercial bovine gelatins was 127.53 and 130.85 s, respectively. The gelling and melting temperatures of FG + GH (19.11 and 21.64, respectively) and CG + GH (19.06 and 20.98, respectively) coatings were significantly lower than that of CG and FG. This reduction in gelling and melting points after addition of GH could be due to the weakening the gel as a result of producing the penetration pores in the infrastructure of gel (Abdelhedi et al., 2019).

### 3.2 Chemical characterization of the coated shrimp

#### 3.2.1 Evaluation of pH

The pH value is an important factor indicating the chemical spoilage of the shrimps, which is frequently related to the quality descent. The changes in pH value during 12 days of storage are recorded in Table 1. As the time growing, the pH value of all treatments rises. The initial pH of all samples on the first day was 6.21, which was reached to above 8.30 in all treatments. Obviously, the pH value in samples treated with gelatin containing GH was continuously increased to above 8.30 at the day 12, but they were still significantly lower than control and also samples treated with gelatin. However, during the storage, the pH of sample treated with FG + GH was significantly lower than that of other samples. The treatment CG + GH was also showed an appropriate as it was significantly affected the pH ascending compared with the FG and CG samples. This is probably due to the antimicrobial effect of GH which blocked the microbial growth.

**Table 1:** Changes in pH during 12 days of storage. Data are reported as mean ± *SD*

Sample	Storage time (day)				
	0	3	6	9	12
Control	6.21 ± 0.05^Ca^	7.54 ± 0.11^Ba^	7.88 ± 0.12^Ba^	8.20 ± 0.05^Aa^	8.71 ± 0.17^Aa^
CG	6.21 ± 0.02^Da^	6.91 ± 0.15^Cb^	7.76 ± 0.14^Bb^	7.95 ± 0.10^Bb^	8.55 ± 0.21^Ab^
FG	6.21 ± 0.05^Ea^	6.92 ± 0.07^Db^	7.30 ± 0.07^Cc^	7.95 ± 0.25^Bb^	8.55 ± 0.29^Ab^
CG + GH	6.20 ± 0.05^Ea^	6.81 ± 0.18^Dc^	7.24 ± 0.19^Cd^	7.63 ± 0.12^Bc^	8.44 ± 0.03^Ac^
FG + GH	6.19 ± 0.06^Ea^	6.75 ± 0.10^Dd^	7.13 ± 0.08^Ce^	7.45 ± 0.25^Bd^	8.31 ± 0.16^Ad^

Note

The different uppercase and lowercase letters show significant difference in rows and columns, respectively.

#### 3.2.2 Free fatty acid determination

The changes in FFA content during 12 days of different treatments are shown in Table 2. The results revealed that adding gelatin hydrolysates to the coating mixture was significantly decreased the FFA content of the samples during 12 days. As it is obvious, the FFA content of the samples coated with CG and FG (0.73 and 0.74, respectively) was not statistically different from the control sample (0.76) until day 3, while from day 6 to 12, they were remarkably lower than the control (*p* < .05). The CG + GH and FG + GH‐treated samples indicated notably lower FFA content than that of CG, FG, and the control from day 3 to 12 (*p* < .05). The lower FFA content in CG and FG samples compared with the control is probably due to the act of the gelatin solutions as a barrier to slow down the hydrolytic rancidity via prevention of the water vapor (Łopusiewicz, Jędra, & Bartkowiak, 2018) and microorganisms with lipolytic activity, as psychrotrophs possess lipases (Saravani, Ehsani, Aliakbarlu, & Ghasempourz, 2019), to the surface of the samples. The effect of CG + GH and FG + GH treatments on the FFA content of samples was significantly higher than CG and FG treatment because of the antioxidant (radical scavenging and inhibition of linoleic acid oxidation) and antimicrobial activities of the gelatin hydrolysates (Data not shown) and this had a synergistic effect on CG and FG coatings.

**Table 2:** Changes in FFA content (in terms of oleic acid in 100 g of sample) during 12 days of storage

Sample	Storage time (day)				
	0	3	6	9	12
Control	0.47 ± 0.01^Ea^	0.76 ± 0.05^Da^	1.76 ± 0.03^Ca^	1.92 ± 0.10^Ba^	2.50 ± 0.08^Aa^
CG	0.47 ± 0.04^Ea^	0.73 ± 0.09^Da^	1.11 ± 0.09^Cb^	1.58 ± 0.07^Bb^	2.36 ± 0.01^Ab^
FG	0.47 ± 0.02^Ea^	0.74 ± 0.00^Da^	1.10 ± 0.05^Cb^	1.51 ± 0.03^Bb^	2.37 ± 0.04^Ab^
CG + GH	0.47 ± 0.00^Da^	0.68 ± 0.01^Cb^	0.99 ± 0.04^Bc^	1.08 ± 0.01^Bc^	1.70 ± 0.09^Ac^
FG + GH	0.46 ± 0.09^Da^	0.65 ± 0.05^Cb^	0.94 ± 0.01^Bc^	1.03 ± 0.03^Bc^	1.68 ± 0.00^Ac^

Note

Data are reported as mean ± *SD*. The different uppercase and lowercase letters show significant difference in rows and columns, respectively.

#### 3.2.3 Total volatile basic nitrogen

The TVB‐N values were increasing during the storage time for all samples (Table 3). The average of acceptable limit for TVB‐N is 30 mg nitrogen per 100 g (Directive E, 2008) which the control sample showed higher amount at 9 days after storage. The TVB‐N of the CG and FG samples were significantly lower than the control after the day 3 of storage and it was acceptable for both of them until day 9. The CG + GH and FG + GH samples showed an acceptable (during 12 days of storage) and notably lower TVB‐N than that of other samples (24.76 and 24.49 mg N/100 g sample, respectively, at day 12 of storage).

**Table 3:** Changes in TVB‐N (mg N/100 g) during 12 days of storage. Data are reported as mean ± *SD*

Sample	Storage time (day)				
	0	3	6	9	12
Control	10.97 ± 0.73^Ea^	18.58 ± 0.45^Da^	25.16 ± 0.66^Ca^	32.03 ± 0.10^Ba^	41.22 ± 0.28^Aa^
CG	10.97 ± 0.41^Ea^	17.59 ± 0.39^Da^	21.58 ± 0.79^Cb^	27.19 ± 0.57^Bb^	33.74 ± 0.76^Ab^
FG	10.96 ± 0.12^Ea^	17.34 ± 0.50^Da^	20.43 ± 0.05^Cb^	25.80 ± 0.83^Bb^	31.50 ± 0.44^Ab^
CG + GH	10.97 ± 0.28^Ca^	15.69 ± 0.61^Bb^	16.11 ± 0.34^Bc^	16.20 ± 0.25^Bc^	24.76 ± 0.98^Ac^
FG + GH	10.90 ± 0.39^Ca^	15.62 ± 0.55^Bb^	16.00 ± 0.11^Bc^	16.15 ± 0.13^Bd^	24.49 ± 0.67^Ac^

Note

The different uppercase and lowercase letters show significant difference in rows and columns, respectively.

Accumulation of TVB‐N has unpleasant influence on properties of fish meat (Li et al., 2012). Concentration of ammonia, dimethylamine, trimethylamine, monoethylamine, and other volatile bases could be a result of bacterial consumption of amino acids in fish muscle, which represents as TVB‐N index (Goulas & Kontominas, 2007). Nitrogen‐containing substances could be catabolized by bacteria resulting in ammonia which can increase the TVB‐N values (Arancibia, Lopez‐Caballero, Gomez‐Guillen, & Montero, 2015).

#### 3.2.4 Lipid oxidation secondary products

The results showed that the malonaldihyde increased during the storage time and the highest malonaldihyde content for all treatments was observed at the day 12 of storage (Table 4). However, at all‐time intervals, the CG + GH and FG + GH treatments led to a significantly lower malonaldihyde content in the samples compared with the other treatments. The increasing of malonaldihyde content in CG and FG treatments was significantly slower than that of control sample except in day 12 of storage (*p* < .05). After 12 days of storage, the malonaldihyde content of the control sample was reached to 2.97 mg/kg, which was significantly higher than CG + GH and FG + GH (0.57 and 0.54 mg/kg, respectively) (*p* < .05). Antioxidant activity of fish gelatin hydrolysates is an important factor in this experiment. This is due to their potential for inhibition of the early steps of lipid oxidation and/or postpone the propagation step (Jridi et al., 2018). Gelatinatious coatings are known to inhibit oxygen from reaching the product and also have antioxidant properties (Farajzadeh, Motamedzadegan, Shahidi, & Hamzeh, 2016). In the present study, the antioxidant effect of gelatin hydrolysates is more significant compared with the gelatin alone.

**Table 4:** Changes in lipid oxidation (mg malonaldihyde/kg) during 12 days of Storage

Sample	Storage time (day)				
	0	3	6	9	12
Control	0.24 ± 0.05^Ea^	0.90 ± 0.02^Da^	1.80 ± 0.21^Ca^	2.65 ± 0.19^Ba^	2.97 ± 0.08^Aa^
CG	0.24 ± 0.00^Ea^	0.78 ± 0.10^Db^	1.02 ± 0.27^Cb^	2.10 ± 0.37^Bb^	2.98 ± 0.00^Aa^
FG	0.24 ± 0.02^Ea^	0.75 ± 0.04^Db^	1.05 ± 0.25^Cb^	2.09 ± 0.13^Bb^	2.90 ± 0.40^Aa^
CG + GH	0.23 ± 0.00^Da^	0.25 ± 0.01^Dc^	0.41 ± 0.01^Cc^	0.48 ± 0.05^Bc^	0.57 ± 0.02^Ab^
FG + GH	0.23 ± 0.01^Ca^	0.24 ± 0.05^Cc^	0.42 ± 0.04^Bc^	0.46 ± 0.03^Bc^	0.54 ± 0.07^Ab^

Note

Data are reported as mean ± *SD*. The different uppercase and lower case letters show significant difference in rows and columns, respectively.

#### 3.2.5 Determination of protein oxidation

**Figure 1:**
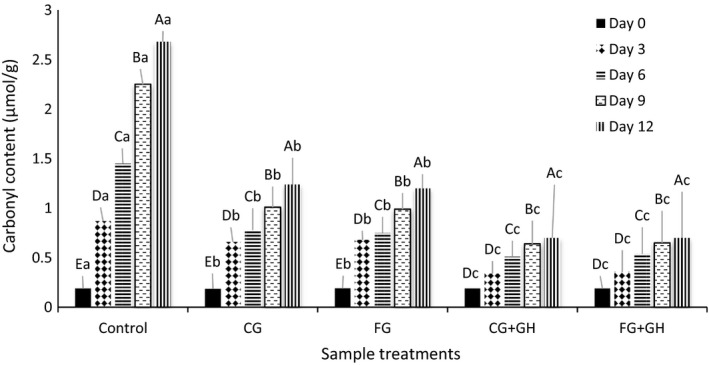
Protein oxidation process of samples during 12 days of storage at 4°C. Data are plotted based on mean values. The different uppercase and lower case letters show significant difference of carbonyl content during storage time and different samples, respectively

The carbonyl content (µmol/g) of all samples was directly related to the storage time Figure 1. Similar results have been achieved when the coated tilapia fillets with fish gelatin incorporated with grape seed extract through vacuum impregnation were stored at 4°C (Zhao et al., 2019). The results of this experiment revealed that the carbonyl content of control sample was 2.68 µmol/g at the day 12, which was significantly higher than CG and FG samples (1.24 and 1.20 µmol/g, respectively, at the day 12) from the day 3 till the last day of storage (*p* < .05). The carbonyl content of FG + GH and CG + GH samples (0.7 µmol/g for both treatments at day 12) was dramatically lower than that of FG and GH and the control sample as well (*p* < .05). This is likely due to the antioxidant effect of fish gelatin hydrolysates in coating during the storage at 4°C. Application of antioxidant agents in coatings is a prominent approach in order to cease the protein oxidation process (Zhang, Li, Jia, Huang, & Luo, 2018). The protein oxidation is an important indicator of structural, nutritional, and functional characteristics of the shrimp samples, which may cause losing water holding ability due to the changes in structure (Feng, Bansal, & Yang, 2016).

### 3.3 Microbial analysis

Due to the high content of free amino acids and nitrogenous substances, fishery products are more susceptible to corruption than red meat. The quality of shrimp decreases dramatically during storage at refrigerator temperatures, as a result of microbial and enzymatic reactions. The effect of different coatings on microbial properties of the samples is shown in Table 5. The initial total count of all samples was 3.88 log CFU/g. All samples total count were significantly increasing during the storage at 4°C (*p* < .05). Among all treatments, the samples coated with CG + GH and FG + GH showed statistically slower rates of total count ascension. The highest total count of 6.74, 6.80, 9.27, 9.32, and 12.97 log CFU/g was observed for FG + GH, CG + GH, FG, CG, and control, respectively, after 12 days of storage at 4°C. As it obvious, only CG + GH and FG + GH coatings could preserve the samples do not cross the maximal permissible total count of raw marine products (7 log CFU/g) (Sallam, Ahmed, Elgazzar, & Eldaly, 2007). The results of the present study showed that the total count of CG, FG, and control samples was passed through 7 log CFU/g at the days of 9, 9, and 6 of storage, respectively, at 4°C. The effect of an edible chitosan‐based coating on microbiological properties of grass carp fillets was investigated and the results showed that the total count of fillets after 15 days of storage at cold temperatures reached to 6.20 log CFU/g, which was significantly lower than that of the control (Yu, Regenstein, Zang, et al., 2018).

**Table 5:** Changes in total microbial count (TC), psychrotrophic count (PC), and lactic acid bacteria count (LAB) (log CFU/g) during 12 days of storage

Analysis	Sample	Storage time (day)				
		0	3	6	9	12
TC	Control	3.88 ± 0.21^Ea^	6.44 ± 0.26^Da^	8.71 ± 0.41^Ca^	9.95 ± 0.78^Ba^	12.97 ± 0.75^Aa^
	CG	3.88 ± 0.09^Ea^	4.75 ± 0.00^Db^	6.21 ± 0.54^Cb^	8.14 ± 0.41^Bb^	9.32 ± 0.64^Ab^
	FG	3.88 ± 0.17^Ea^	4.70 ± 0.58^Db^	6.18 ± 0.57^Cb^	8.20 ± 0.33^Bb^	9.27 ± 0.36^Ab^
	CG + GH	3.88 ± 0.04^Ca^	3.89 ± 0.31^Cc^	3.97 ± 0.13^Cc^	5.34 ± 0.90^Bc^	6.80 ± 0.22^Ac^
	FG + GH	3.87 ± 0.12^Ca^	3.89 ± 0.09^Cc^	3.99 ± 0.24^Cc^	5.33 ± 0.56^Bc^	6.74 ± 0.74^Ac^
PC	Control	2.94 ± 0.72^Ea^	4.86 ± 0.35^Da^	6.59 ± 0.44^Ca^	7.95 ± 0.86^Ba^	8.31 ± 0.85^Aa^
	CG	2.94 ± 0.49^Ea^	3.45 ± 0.00^Db^	4.75 ± 0.39^Cb^	6.38 ± 0.56^Bb^	6.90 ± 0.59^Ab^
	FG	2.94 ± 0.65^Ea^	3.40 ± 0.37^Db^	4.78 ± 0.37^Cb^	6.35 ± 0.50^Bb^	6.97 ± 0.49^Ab^
	CG + GH	2.89 ± 0.21^Ba^	3.09 ± 0.14^Bc^	3.17 ± 0.13^Bc^	4.54 ± 0.30^Ac^	4.76 ± 0.62^Ac^
	FG + GH	2.87 ± 0.19^Ba^	3.04 ± 0.01^Bc^	3.10 ± 0.24^Bc^	4.58 ± 0.62^Ac^	4.74 ± 0.384^Ac^
LAB	Control	2.55 ± 0.41^Ea^	4.02 ± 0.47^Da^	4.82 ± 0.59^Ca^	5.99 ± 0.56^Ba^	6.75 ± 0.43^Aa^
	CG	2.55 ± 0.27^Ea^	3.06 ± 0.39^Db^	4.19 ± 0.47^Cb^	5.65 ± 0.63^Bb^	6.00 ± 0.72^Ab^
	FG	2.55 ± 0.36^Ea^	3.13 ± 0.51^Db^	4.12 ± 0.55^Cb^	5.63 ± 0.44^Bb^	6.07 ± 0.79^Ab^
	CG + GH	2.55 ± 0.54^Ca^	2.64 ± 0.27^Cc^	2.67 ± 0.49^Cc^	3.36 ± 0.58^Bc^	4.34 ± 0.65^Ac^
	FG + GH	2.53 ± 0.29^Ca^	2.59 ± 0.46^Cc^	2.61 ± 0.36^Cc^	3.40 ± 0.56^Bc^	4.31 ± 0.584^Ac^

Note

Data are reported as mean ± *SD*. The different uppercase and lowercase letters show significant difference in rows and columns for each analysis, respectively.

Similar to the TC, the PC results showed uptrend during 12 days of storage at 4°C Table 5. The CG and FG treatments led to a significant reduction in the PC (1.41 and 1.34 log CFU/g, respectively) compared with the control sample at the day 12 of storage, while the CG + GH and FG + GH treatments reduced the PC (3.55 and 3.57 log CFU/g) in comparison with control remarkably. The maximal permissible PC is 7 log CFU/g (Goulas & Kontominas, 2005) which shows that the control sample at the day 6 of storage is very close to this amount (6.59 log CFU/g), whereas the CG, FG, CG + GH, and FG + GH samples could retain below this value till the day 12 of storage. However, it seems that the CG and FG samples would pass through the acceptable value if the storage time was longer than 12 days.

In LABs assay, the microbial count was ascending during the storage‐like TC and PC (Table 5). The initial count for samples was 2.55 log CFU/g, which was increased for all of them until the last day of storage. Similar results were observed during the storage of grass carp wrapped with chitosan‐based coatings (Yu, Regenstein, Zang, et al., 2018). After 12 days of storage, the number of LABs of control sample was reached to 6.75 log CFU/g which was not significantly higher than that in CG and FG (6.60 and 6.67 log CFU/g, respectively). CG + GH and FG + GH samples were markedly decreased the rate of LABs growth as the number of these bacteria at the last day of storage (4.34 and 4.31 log CFU/g, respectively) was lower than even CG and FG samples. After all, the antibacterial effect of the GH may be the reason for these results and the inhibition effect of CG/FG coatings on microbial growth is mainly due to their barrier effect. This can be concluded because the LAB, microaerophilic bacteria, growth was not significantly lower than the control, where in the TC and PC results were the opposite.

### 3.4 Sensorial analysis of coated shrimp

The color, odor, texture, and finally the total acceptance of the samples were slumped during the 12 days of storage at 4°C (Table 6). It is clear that coating the shrimps with CG and FG resulted in significantly better sensorial properties after the storage time, but in all cases, the CG and FG samples were acceptable (score was ≥ 3) till the day 6 of storage. The texture of control sample was the only characteristic, which was acceptable after 6 days, while other property scores were reached to below 3 because off‐odor and off‐color appeared. After day 6, the slime layer was observed by all the panelists on the CG, FG, and control samples, but the slime and elasticity of CG and FG were lower. However, the off‐odor was the first sign of spoilage for all the panelists and the off‐color was the second sign convenient to detect during the storage. The CG + GH and FG + GH samples showed a significantly positive effect on all sensorial properties compared with the other samples where all the characteristics were acceptable after 12 days of storage. The total acceptance of the control, CG, FG, CG + GH, and FG + GH samples were 3, 6, 6, 12, and 12 days, respectively, suggesting that a better preservation was performed when the GH was added to the coatings. These results were in agreement with changes in microbial, TVB‐N, lipid oxidation, and other analyses.

**Table 6:** Effect of storage (0–12 days) at 4°C on sensory properties. Data are reported as mean ± *SD*

Sensory characteristics	Sample	Storage time (day)				
		0	3	6	9	12
Color	Control	5.00 ± 0.00^Aa^	4.10 ± 0.71^Bb^	2.60 ± 0.55^Cc^	2.00 ± 0.26^Dc^	1.00 ± 0.00^Ec^
	CG	5.00 ± 0.00^Aa^	4.33 ± 0.45^Bb^	3.60 ± 0.57^Cb^	2.83 ± 0.57^Db^	1.60 ± 0.54^Eb^
	FG	5.00 ± 0.00^Aa^	4.30 ± 0.54^Bb^	3.59 ± 0.84^Cb^	2.80 ± 0.43^Db^	1.66 ± 0.86^Eb^
	CG + GH	5.00 ± 0.00^Aa^	4.80 ± 0.90^Ba^	3.80 ± 0.59^Ca^	3.60 ± 0.74^Ca^	3.10 ± 0.35^Da^
	FG + GH	5.00 ± 0.00^Aa^	4.81 ± 0.56^Ba^	3.83 ± 0.61^Ca^	3.66 ± 0.51^Ca^	3.20 ± 0.42^Da^
Odor	Control	5.00 ± 0.00^Aa^	3.83 ± 0.16^Bc^	2.80 ± 0.39^Cc^	1.83 ± 0.56^Dc^	1.00 ± 0.00^Ec^
	CG	5.00 ± 0.00^Aa^	4.50 ± 0.18^Bb^	3.1 ± 0.26^Cb^	2.80 ± 0.00^Db^	2.00 ± 0.00^Eb^
	FG	5.00 ± 0.00^Aa^	4.50 ± 0.27^Bb^	3.3 ± 0.00^Cb^	2.66 ± 0.24^Db^	2.00 ± 0.00^Eb^
	CG + GH	5.00 ± 0.00^Aa^	4.80 ± 0.90^Ba^	4.66 ± 0.29^Ca^	3.33 ± 0.17^Da^	3.30 ± 0.60^Da^
	FG + GH	5.00 ± 0.00^Aa^	4.81 ± 0.56^Ba^	4.83 ± 0.65^Ca^	3.60 ± 0.50^Da^	3.31 ± 0.33^Da^
Texture	Control	5.00 ± 0.00^Aa^	4.1 ± 0.52^Bc^	3.20 ± 0.27^Cc^	1.66 ± 0.63^Dc^	1.00 ± 0.00^Ec^
	CG	5.00 ± 0.00^Aa^	4.66 ± 0.45^Bb^	3.83 ± 0.25^Cb^	2.60 ± 0.45^Db^	2.00 ± 0.00^Eb^
	FG	5.00 ± 0.00^Aa^	4.66 ± 0.54^Bb^	3.66 ± 0.59^Cb^	2.66 ± 0.50^Db^	2.00 ± 0.00^Eb^
	CG + GH	5.00 ± 0.00^Aa^	5.00 ± 0.00^Aa^	4.83 ± 0.36^Aa^	3.66 ± 0.66^Ba^	3.60 ± 0.58^Ba^
	FG + GH	5.00 ± 0.00^Aa^	5.00 ± 0.00^Aa^	4.83 ± 0.30^Aa^	3.66 ± 0.27^Ba^	3.60 ± 0.55^Ba^
Total acceptance	Control	5.00 ± 0.00^Aa^	4.1 ± 0.20^Bc^	2.30 ± 0.41^Cc^	2.1 ± 0.63^Dc^	1.00 ± 0.00^Ec^
	CG	5.00 ± 0.00^Aa^	4.30 ± 0.55^Bb^	3.1 ± 0.50^Cb^	2.60 ± 0.55^Db^	2.00 ± 0.20^Eb^
	FG	5.00 ± 0.00^Aa^	4.40 ± 0.33^Bb^	3.00 ± 0.00^Cb^	2.66 ± 0.25^Db^	2.10 ± 0.22^Eb^
	CG + GH	5.00 ± 0.00^Aa^	4.80 ± 0.20^Ba^	4.41 ± 0.55^Ca^	3.66 ± 0.54^Da^	3.00 ± 0.00^Ea^
	FG + GH	5.00 ± 0.00^Aa^	4.81 ± 0.45^Ba^	4.40 ± 0.40^Ca^	3.60 ± 0.18^Da^	3.10 ± 0.16^Ea^

Note

The different uppercase and lowercase letters show significant difference in rows and columns in a same characteristic, respectively.

### 3.5 Shelf‐life extension capability

**Figure 2:**
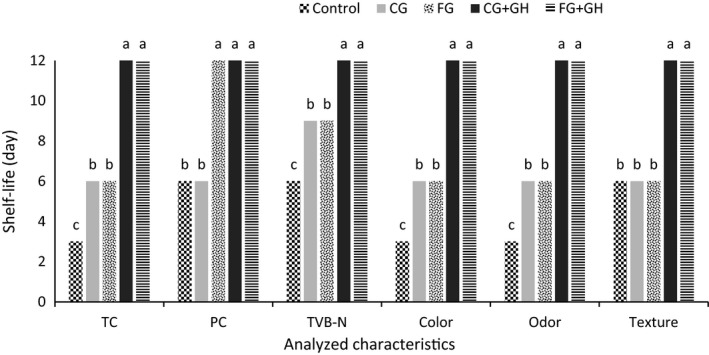
Shelf‐life extension of samples at 4°C. Total microbial count (TC), Psychrotroph count (PC), Total volatile basic nitrogen (TVB‐N). Data are plotted based on mean values. The different lower case letters show significant difference in each characteristic

The ability of different coatings for extension of the shrimp shelf life is shown in Figure 2. As it can be seen, the coatings containing gelatin hydrolysates had significant effect on shelf‐life extension in chemical, microbial, and sensorial properties of samples during 12 days of storage at 4°C. The gelatin coatings with lack of hydrolysates showed better preservation than the control sample in all parameters except for the texture. Maximum shelf life which could be accepted for control samples were 6 days though the maximum preservation capability of CG and FG samples was 9 days. These results showed that the GH were significantly improved the shelf‐life extension capability of coatings.

## 4 CONCLUSIONS

Gelatin characterization indicated that the fish (*Scomberomorus commerson*) gelatin is an appropriate replacement for commercial bovine gelatin for many applications including active coating agents. The overall results of this study illustrated that the application of fish gelatin hydrolysates as an active ingredient in gelatin solutions is a well‐defined approach for using these antimicrobial and antioxidant agents for food preservation methods. In addition, the treatments containing CG compared with those containing FG showed no significant different effect on most of quality factors including pH value, FFA content, TVB‐N, lipid oxidation, microbial count, and also sensorial analysis, which proves that fish gelatin can be a proper substitution for commercial bovine gelatin for fabrication of edible active coatings.

## CONFLICT OF INTEREST

The authors declare that they do not have any conflict of interest.

## ETHICAL APPROVAL

This study does not involve any human or animal testing.

## INFORMED CONSENT

Written informed consent was obtained from all study participants.
